# Subacute Hypoxia Induces Cardiac Remodeling and Mitochondrial Dysfunction via Apoptotic Pathways in a Rabbit Model of Tracheal Stenosis

**DOI:** 10.3390/jcdd12100377

**Published:** 2025-09-24

**Authors:** Taeyun Kim, Kyoung-Im Cho, Hyoung Kyu Kim, Chulho Oak, Jin Han, Hyoung Shin Lee, Yohan Jeon

**Affiliations:** 1 Department of Internal Medicine, Kosin University College of Medicine, Busan 49267, Republic of Korea; 2 Basic Research Laboratory, Cardiovascular and Metabolic Disease Core Research Support Center, Inje University, Busan 47329, Republic of Korea; phyhanj@inje.ac.kr; 3 Department of Otolaryngology-Head and Neck Surgery, Kosin University College of Medicine, Busan 49267, Republic of Korea; hsleeent@gmail.com; 4 Department of Pathology, Kosin University College of Medicine, Busan 49267, Republic of Korea; han0328_j@naver.com

**Keywords:** tracheal stenosis, hypoxia, cardiac remodeling, mitochondria, apoptosis

## Abstract

Myocardial hypoxia is a major cause of cardiac dysfunction, triggering cellular injury and apoptosis. This study aims to investigate the effects of subacute hypoxia on cardiac remodeling and mitochondrial oxygen consumption. This study is based on a rabbit experimental model. Hypoxia was induced using a rabbit tracheal stenosis model. Endotracheal intubation with a 1.5 cm segmented tube wrapped with an absorbable hemostat was used to generate tracheal stenosis in six rabbits. Sham controls (*n* = 3) underwent tracheotomy, with the tracheal exposure site being sutured immediately. After 1 week, the tube was removed. Echocardiography and mitochondrial function from both groups were morphologically and functionally analyzed at 2 weeks after endoscopic confirmation of tracheal stenosis. Compared to sham group, tracheal stenosis group showed significantly reduced interventricular septal wall thickness (2.3 ± 0.1 mm vs. 2.7 ± 0.2 mm, *p* = 0.08) and enlarged left ventricular end-diastolic volume (5.86 ± 0.58 mL vs. 5.39 ± 0.18 mL, *p* = 0.46) with reduced left ventricular ejection fraction (54.5 ± 5.3% vs. 66.9 ± 4.0%, *p* = 0.005). The tracheal stenosis group showed significantly reduced mitochondrial oxygen consumption at state 3 with reduced respiratory control ratio. Caspase activities (caspase-9 and caspase-3) were increased in the tracheal stenosis group than in the sham group. Subacute hypoxia induced by the tracheal stenosis model causes cardiac remodeling and mitochondrial dysfunction through apoptotic pathways. This study suggests that management of hypoxia could prevent cellular apoptosis and cardiac dysfunction.

## 1. Introduction

Hypoxia, a condition characterized by inadequate oxygen supply to tissues, is a significant pathophysiological factor contributing to various cardiovascular diseases [1]. Prolonged hypoxia can result in cellular injury, apoptosis, and alterations in mitochondrial function, all of which play critical roles in the progression of cardiac dysfunction [1]. The heart, being highly dependent on oxygen for its metabolic processes, is particularly vulnerable to the effects of hypoxia. Severe hypoxic conditions can lead to cardiac remodeling, reduced contractility, and impaired cardiac output, ultimately contributing to heart failure [1,2,3,4,5].

While previous studies have predominantly focused on the consequences of acute or chronic ischemic injury [1,2,3,4,5,6,7], there remains a lack of understanding regarding the morphological and biological changes occurring during the subacute phase [8]. This transitional period could be important for identifying early markers of cardiac dysfunction and developing targeted interventions.

Tracheal stenosis is a condition that can cause respiratory compromise and hypoxia, and it could provide a practical model for studying hypoxia-related pathophysiology. Rabbit models of tracheal stenosis have been widely utilized due to the histological similarity of the rabbit trachea to that of humans and its comparable size and complexity to those of a human newborn [9,10,11]. The rabbit tracheal stenosis model has been shown to induce hypoxic conditions and is therefore useful for studying airway-related physiological and pathological processes [12,13]. Our previous research with an endotracheal intubation with a segmented tube showed the feasibility of this model by examining the histological and physiological changes induced by tracheal stenosis following endotracheal intubation [10,14]: this approach induced consistent and precise mucosal injury while minimizing excessive tissue damage to the cartilage [10].

In this context, we hypothesized that prolonged airway obstruction would result in systemic hypoxia, which in turn would lead to myocardial remodeling and mitochondrial dysfunction through impaired oxygen utilization and activation of apoptotic pathways. This study expands on the previous experiment by evaluating the impact of continuous, subacute hypoxic conditions on cardiac function and mitochondrial oxygen consumption using a rabbit tracheal stenosis model induced by endotracheal intubation with a 1.5 cm segmented tube wrapped in an absorbable hemostat.

## 2. Materials and Methods

### 2.1. Study Design

All animal procedures were conducted in accordance with the guidelines published in the Guide for the Care and Use of Laboratory Animals (DHEW publication NIH 85–23, revised 2010, Office of Science and Health Reports, DRR/NIH, Bethesda, MD, USA). Animal experiments in this study were performed in full accordance with the ARRIVE guideline reporting guidelines. The study protocol was approved by the Committee on Animal Research of the College of Medicine at Kosin University (Approval no: KMAP-16-11). Nine male New Zealand white rabbits (Taesung Laboratory Animal Science, Busan, Korea), each weighing between 3.0 and 3.7 kg, were used in the experiment. Three rabbits underwent sham surgery to assess the effects of tracheostomy, while a segmented tracheal tube was intubated into the remaining six rabbits. After all experiments, rabbits were first anesthetized using an intramuscular injection of ketamine (35 mg/kg) and xylazine (5 mg/kg). Once deep anesthesia was confirmed by the absence of a pedal withdrawal reflex, euthanasia was performed using carbon dioxide inhalation in a closed chamber, in accordance with institutional animal care guidelines.

### 2.2. Procedure for Inducing Tracheal Stenosis

The rabbits were anesthetized intramuscularly with ketamine (35 mg/kg) and xylazine (5 mg/kg). Each was positioned supine on a heated operating table, with body temperature maintained at 39 °C by monitoring rectal temperature. Heart rate and respiratory rate were continuously observed. For additional analgesia, 2 mL of 1% lidocaine hydrochloride was injected subcutaneously into the anterior neck. The area was shaved and disinfected before surgery. A midline vertical incision was made on the anterior neck to expose the trachea and larynx. Minimal bleeding occurred due to the avascular dissection plane between the strap muscles. A transverse incision was then created on the anterior tracheal wall between the fifth and sixth tracheal rings, extending two-thirds of the circumference. For sham models (*n* = 3), the tracheal incision site was closed using a single 4-0 Vicryl suture.

**Figure 1:**
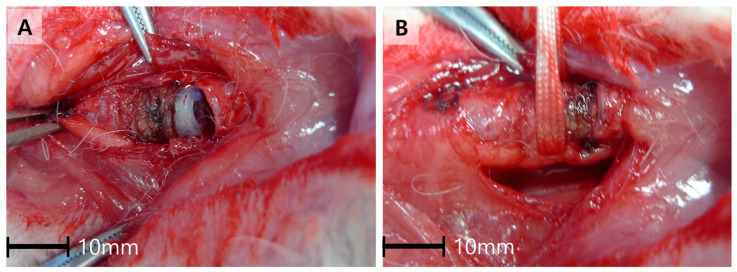
Insertion of a segmented endotracheal tube into the trachea through the tracheostomy site. (**A**) Placement of a segmented endotracheal tube wrapped with absorbable hemostat into the tracheal lumen. (**B**) Tracheal incision closed after tube placement, showing appropriate positioning of the tube within the trachea.

A segmented pediatric intubation tube (3.5 mm inner diameter, Covidien, Mansfield, MA, USA) measuring 1.5 cm in length was wrapped with a commercial absorbable hemostat, Surgicel (Ethicon, Cincinnati, OH, USA), and inserted into the trachea through the tracheostomy site (Figure 1). The amount of Surgicel was adjusted to ensure the tube adequately filled the tracheal lumen, providing appropriate pressure on the tracheal mucosa. The tracheal rings were aligned in their anatomical position, and the incision was closed using a single 4-0 silk interrupted suture. The strap muscles and skin were sutured using the same technique.

### 2.3. Assessment of Tracheal Stenosis

**Figure 2:**
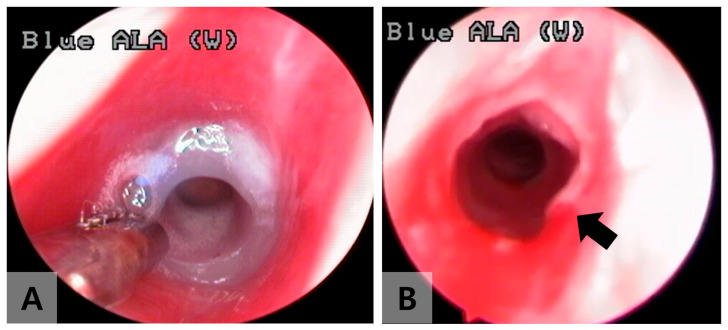
An image 1 week after segmented endotracheal tube insertion (**A**) and an image just right after the tube removal (**B**). A mild degree of circumferential granulation tissue is noted at the 5 o’clock position on the right-sided image (black arrow).

**Figure 3:**
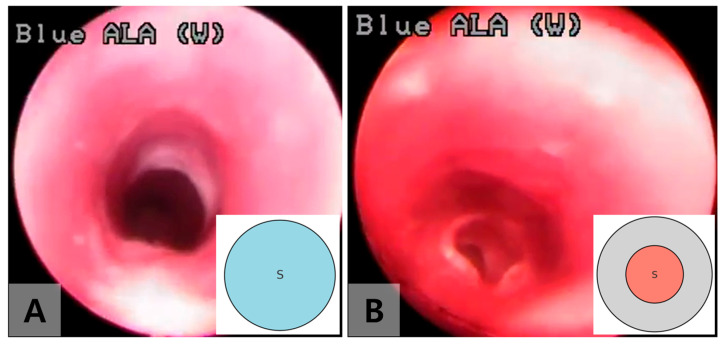
Endoscopic images showing the progression of tracheal stenosis following tube removal. (**A**) Approximately 55% tracheal stenosis was observed 1 week after tube removal. (**B**) Approximately 84% tracheal stenosis was observed 2 weeks after tube removal.

One week after tube insertion, the tubes were removed transorally under bronchoscopy (Figure 2). Endoscopic evaluations were performed under identical anesthetic conditions at 1 and 2 weeks after the tube removal to assess the degree of tracheal stenosis (Figure 3). Three weeks post-surgery, the rabbits were sacrificed, and the excised tracheal tissue was processed for standard hematoxylin and eosin staining and examined under a microscope. Similarly, three sham models underwent endoscopic evaluation after tracheostomy following the same protocol as the experimental group, and their tracheal tissue was histologically analyzed 3 weeks after the tracheostomy.

The degree of stenosis was quantified using bronchoscopic images taken at each time point, based on an area-based index. It was calculated as (1 − s/S) × 100, where s represents the intraluminal area of the trachea after tube removal, and S represents the corresponding area before tube insertion at the level of the sixth tracheal ring (Figure 3). Measurements were performed digitally using Adobe Photoshop CS3 Software (Adobe Systems Inc., San Jose, CA, USA), based on pixel dimensions. The region of interest was visually identified and manually traced along the inner margin of the tracheal lumen using the freehand tool. To complement this quantitative approach, stenosis severity was also classified according to the Myer—Cotton grading system: Grade I (<50% obstruction), Grade II (51–70%), Grade III (71–99%), and Grade IV (no detectable lumen). All measurements were performed twice by the same investigator, and mean values were used to minimize intra-observer variability.

### 2.4. Measurement of Mitochondrial Oxygen Respiration and Apoptotic Activity

Mitochondrial oxygen consumption was assessed using an Oxygraph-2k (Oroboros, Innsbruck, Austria) in a 2000 μL air-saturated chamber at room temperature. Excised left ventricular heart tissue was utilized. The respiration medium contained 145 mmol/L KCl, 30 mmol/L HEPES, 5 mmol/L KH_2_PO_4_, 3 mmol/L MgCl_2_, 0.1 mmol/L EGTA, and 0.1% BSA (pH 7.4). State 4 respiration was measured using 5 mmol/L glutamate/malate as substrates, while state 3 respiration (active respiration) was induced by adding 0.5 mmol/L ADP. Oxygen consumption was expressed in picomoles of O_2_ per second per milligram of protein. The respiratory control ratio (RCR) was calculated as the ratio of state 3 (ADP-stimulated respiration) to state 4 (resting respiration after ADP depletion [15]. This value reflects the maximum increase in mitochondrial oxygen usage that exceeds the baseline oxygen required to compensate for proton leak in the Electron Transport Chain. A high RCR signifies that mitochondria exhibit a strong capacity for phosphorylation-driven respiration compared to the respiration needed to counteract proton leak [15,16].

In addition, to evaluate apoptosis-associated mitochondrial dysfunction in the left ventricular myocardium under tracheal stenosis-induced hypoxia conditions, we measured the activities of caspase-3/7 and caspase-9 using the Caspase-Glo^®^ 3/7 Assay Kit and Caspase-Glo^®^ 9 Assay Kit (Promega, Madison, WI, USA), according to the manufacturer’s protocols. After euthanasia, whole hearts were rapidly extracted from both the tracheal stenosis group and the sham group. Approximately 50 mg of left ventricular tissue was excised from each heart and homogenized in ice-cold cell lysis buffer (provided in the kit) using a Dounce homogenizer. The homogenates were incubated on ice for 15 min and then centrifuged at 12,000× *g* for 10 min at 4 °C to collect the supernatant. Protein concentration was determined using the bicinchoninic acid (BCA) protein assay. For each assay, 100 μg of total protein per sample was transferred into a 96-well white-walled plate in a total volume of 50 μL. An equal volume (50 μL) of Caspase-Glo^®^ reagent (either 3/7 or 9) was added to each well. The plate was gently mixed and incubated at room temperature for 1 h in the dark to allow complete caspase reaction and stabilization of the luminescent signal. Luminescence was measured using a microplate luminometer. Caspase activity was expressed as relative luminescence units (RLU) and normalized to total protein content. All measurements were performed in triplicate.

### 2.5. Echocardiography

Echocardiographic assessments and measurements were conducted following established protocols for small animals [17]. Standard transthoracic 2D imaging, M-mode, and Doppler flow analyses were performed with continuous ECG monitoring. The examinations were carried out by an expert cardiologist, KI Cho. The changes in values between baseline and 2 weeks after tube removal were compared between the experimental group and the sham group. Representative images of the experimental rabbit are shown in Supplementary Figures S1 and S2.

### 2.6. Statistical Analysis

All numerical data are presented as mean ± standard deviation (SD). Given the small sample sizes (*n* = 6 in the experimental group and *n* = 3 in the sham group), comparisons between the two groups were performed using an unpaired two-tailed Student’s *t*-test. Statistical analyses were performed using SPSS version 25.0 (SPSS Inc., Chicago, IL, USA). A *p* < 0.05 was considered statistically significant.

## 3. Results

A surgical procedure involving tube insertion through the tracheostomy site was performed on 6 rabbits in the experimental group and 3 in the sham group, with no significant bleeding observed. Surgicel was applied around the tube one to three times (median: twice), depending on the size of the tracheal lumen, to securely fix the tube within the trachea. The entire procedure lasted about 10 min. None of the 9 rabbits died during the observation period, and all were sacrificed 3 weeks after the tube insertion (e.g., 2 weeks following tube removal).

**Figure 4:**
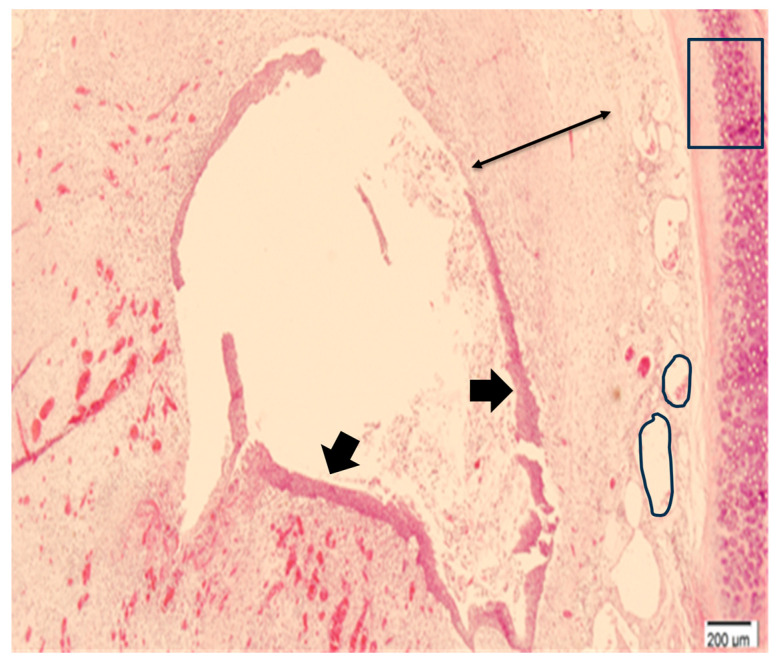
Histological examination 2 weeks after tube removal: The thick black arrow indicates the epithelium, which shows squamous metaplasia. The thin black arrow points to thickening of the lamina propria due to fibrosis. The circled area highlights a blood vessel, representing neovascularization within the fibrotic tissue.

Tracheal stenosis was observed in all rabbits in the experimental group, showing circumferential granulation tissue (Table 1, Figure 2 and Figure 3). Approximately 55% stenosis was identified 1 week after tube removal, and 84% stenosis was found 2 weeks after tube removal (Figure 3). Histological examination similarly showed narrowing of the tracheal lumen, squamous metaplasia of mucosal epithelium, thickening and fibrosis of lamina propria, and neovascularization after 2 weeks of tube removal (Figure 4). In contrast, the histologic finding of the sham model showed a normal tracheal wall with mild mesothelial reaction at the site of tracheostomy.

Echocardiography at baseline and the tracheal stenosis model exhibited significantly lower left ventricular ejection fraction compared to the sham group (54.5 ± 5.3% vs. 66.9 ± 4.0%, *p* = 0.005). The tracheal stenosis model exhibited a trend toward reduced interventricular septal wall thickness (2.3 ± 0.1 vs. 2.7 ± 0.2 mm, *p* = 0.08) and increased left ventricular end-diastolic volume (5.86 ± 0.58 vs. 5.39 ± 0.18 mL, *p* = 0.46) compared to the sham group, though these differences did not reach statistical significance.

**Figure 5:**
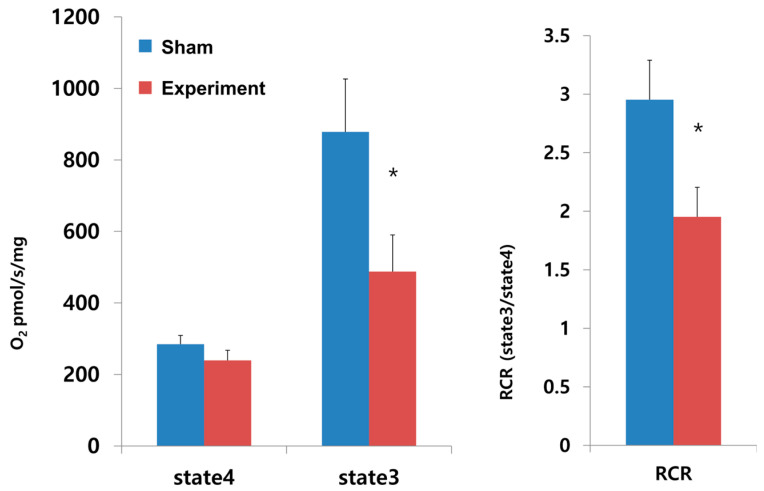
Mitochondrial oxygen consumption (O_2_ pmol/s/mg) and respiratory control rate (RCR) in the sham group and experiment group. An asterisk denotes statistical significance at *p* < 0.05.

**Figure 6:**
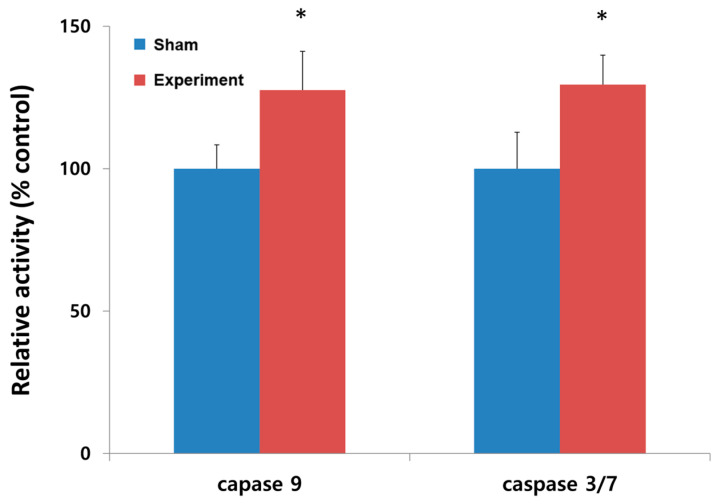
Relative caspase activity (% control) in the sham group and experiment group. An asterisk denotes statistical significance at *p* < 0.05.

The tracheal stenosis model showed significantly reduced mitochondrial oxygen consumption at state 3 with reduced respiratory control ratio (Figure 5). The mean value of mitochondrial oxygen consumption at state 3 was 875 pmol/s/mg in the experimental group versus 480 pmol/s/mg in the sham group (*p* < 0.05). The respiratory control rate was 2.9 ± 0.3 in the sham group and 1.9 ± 0.2 in the experiment group (*p* < 0.05). On the caspase activity assay, the tracheal stenosis model showed increased hypoxia-induced apoptosis via caspase-9 and caspase-3/-7 apoptotic pathway (Figure 6). Compared to the sham group, the activity of caspase 9 and that of caspase 3 increased 30% over baseline levels.

## 4. Discussion

This experimental study found that subacute hypoxia induced by a rabbit tracheal stenosis model leads to significant cardiac remodeling and mitochondrial dysfunction. Echocardiography performed 2 weeks after tube removal revealed a marked reduction in systolic function. Additionally, mitochondria in the tracheal stenosis model exhibited impaired ATP generation efficiency, indicative of oxidative phosphorylation dysfunction. Activation of the intrinsic apoptotic pathway was also confirmed. These findings provide valuable insights into the pathophysiological mechanisms linking subacute hypoxic conditions, cardiac dysfunction, and mitochondrial impairment, underscoring the importance of addressing the transitional phase between acute and chronic hypoxic injury to prevent progressive damage.

Our results showed that the group with tracheal stenosis experienced marked changes in cardiac structure and function compared to the sham group. Specifically, the reduction in interventricular septal wall thickness, enlargement of the LV end-diastolic volume, and reduction in LV ejection fraction suggest that prolonged subacute hypoxia induces pathological remodeling of the myocardium. These results expand the previous experimental studies demonstrating that acute ischemia triggers ventricular dilatation, thinning of myocardial walls, and systolic dysfunction [18]. Echocardiography-proven myocardial structural change in the subacute phase could further link chronic ischemic insult leading to heart failure [19].

Intrinsic mitochondrial apoptotic analysis revealed reduced oxygen consumption during state 3 respiration and a decreased respiratory control ratio in the tracheal stenosis group. These findings suggest that mitochondria function suboptimally under subacute hypoxic conditions, likely due to limited oxygen availability, leading to reduced electron transport chain efficiency. Mitochondria play a pivotal role in cellular energy metabolism, and their dysfunction under hypoxic conditions can lead to energy deficits, increased reactive oxygen species production, and the activation of apoptotic pathways [4,6,7]. In this study, elevated caspase-9 and caspase-3 activities in the tracheal stenosis group further confirm the activation of intrinsic apoptotic pathways in rabbits damaged by subacute hypoxia. The link between mitochondrial dysfunction and apoptosis aligns with prior research indicating that hypoxia-induced mitochondrial damage initiates cascades leading to cardiomyocyte death. This is consistent with recent studies showing that mitochondrial dysfunction under hypoxia can activate apoptotic signaling even before overt cell death is evident. For instance, the expression of Gadd45β protein is upregulated by hypoxia, which promotes apoptosis in cardiomyocytes. Suppression of Gadd45β expression has been shown to reduce hypoxia-induced cell death [20]. Another in vivo study showed that the activation of AMP-activated protein kinase plays a crucial role in reducing hypoxia/reoxygenation-induced apoptosis in cardiomyocytes, which suggests that AMP-activated protein kinase can suppress cell death by regulating intracellular stress responses [21]. These studies implicate that hypoxia induces apoptosis in cardiomyocytes and that modulation of specific proteins or pathways can attenuate these effects, further strengthening our interpretation that the observed caspase activation reflects early hypoxia-induced stress responses, which may evolve into irreversible myocardial damage over time if unaddressed. However, it should be noted that while increased caspase activity is suggestive of intrinsic apoptotic pathway activation, it does not directly confirm irreversible cardiomyocyte loss. Cells under subacute hypoxic stress may activate adaptive mechanisms, such as the hypoxia-inducible factor cascade, to prevent progression to cell death. Therefore, further studies using direct markers of apoptosis or necrosis are needed to delineate the extent of actual cardiomyocyte death and its clinical implications.

The rabbit tracheal stenosis model employed in this study could effectively mimic hypoxia-induced cardiac dysfunction observed in clinical scenarios. The use of a segmented tube to induce tracheal stenosis ensures consistent and controlled hypoxic conditions, closely resembling the pathophysiology of prolonged endotracheal intubation in humans [10,14]. Compared to more invasive or chemically induced models, this method preserves physiologic relevance while minimizing extensive tissue damage. Additionally, the gradual development of hypoxia in this model allows for the investigation of subacute changes, a phase that has been understudied in existing literature.

The findings of this study have several clinical implications. First, the observed link between subacute hypoxia and mitochondrial dysfunction underscores the potential for therapeutic strategies targeting mitochondrial preservation. Agents that enhance mitochondrial function or prevent the activation of apoptotic pathways, such as mitochondrial antioxidants or inhibitors of caspase activity, may hold promise for mitigating hypoxia-induced cardiac injury [22]. Second, the identification of cardiac remodeling during the subacute phase highlights the importance of early detection and intervention. Echocardiographic parameters such as LV wall thickness and ejection fraction could serve as valuable markers for monitoring cardiac health in patients experiencing hypoxia. Third, despite the limited sample size, the absence of clinical evidence directly linking tracheal stenosis to chronic hypoxemia emphasizes the importance of future research involving systemic oxygenation assessments to elucidate the potential relationship between prolonged airway obstruction and myocardial hypoxia. Fourth, apoptosis was assessed only by luminescence-based caspase activity assays, without histological staining of cardiac tissue. Therefore, our findings cannot distinguish whether the increased caspase activity reflects upregulation within individual cardiomyocytes or a greater number of caspase-positive cells, nor can they demonstrate the spatial distribution of apoptotic cells within the left ventricle.

While the rabbit tracheal stenosis model offers significant advantages, there are limitations to consider. First, the small sample size may limit the generalizability of the findings, and the relatively short observation period may not capture long-term outcomes of hypoxia-induced cardiac dysfunction. The number of animals was determined based on prior pilot data and institutional ethical guidelines to minimize animal use, rather than on a priori power analysis. While this approach was approved by the institutional review committee, it nonetheless represents a limitation of the study. Future studies with larger sample sizes and formal power calculations will be required to validate and extend these observations. Second, the study did not assess systemic inflammatory responses or neurohormonal changes that may contribute to hypoxic cardiac injury. Future studies should explore these aspects and investigate the efficacy of potential therapeutic interventions in mitigating mitochondrial dysfunction and cardiac remodeling. Third, this study lacks phenotypic data confirming myocardial hypoxia. While mitochondrial dysfunction and apoptotic signaling suggest hypoxia-related mechanisms, direct measurements such as myocardial tissue oxygenation levels or arterial blood gas analysis were not performed due to ethical and technical constraints. Furthermore, we did not systematically collect physiological monitoring data from the animals prior to sacrifice, particularly parameters that might have differed between those with or without significant cardiac tissue activity. Instead, progressive body weight loss and the presence of stridor were observed as the reliable indicators before sacrifice, but future studies incorporating more comprehensive physiological monitoring may provide valuable early markers of subacute hypoxia-induced cardiac injury. Fourth, this experiment lacks direct measurements of total ATP levels, which could have provided more conclusive evidence for mitochondrial dysfunction under subacute hypoxic conditions. Future investigations should incorporate such analyses to more comprehensively assess cellular energy status. Fifth, this study could not establish a causal relationship between subacute hypoxia and the observed cardiac and mitochondrial dysfunction. Although our findings suggest a strong association, potential contributions from other systemic stress responses cannot be fully excluded. Future studies employing pharmacologic or genetic modulation of hypoxia signaling pathways (e.g., HIF-1α inhibition) would be of importance to clarify the causality. Sixth, the absence of a systemic hypoxia group (e.g., reduced FiO_2_), through which the isolated effects of hypoxia on physiological impacts could be measured, limits our ability to fully distinguish the effects of hypoxia from those related to mechanical airway obstruction. Seventh, our model induces sustained hypoxia, whereas clinical conditions like obstructive sleep apnea or chronic obstructive pulmonary disease often involve intermittent hypoxia. Although our approach helped isolate direct hypoxic effects, it may not fully capture the impact of repeated desaturation-reoxygenation. Future studies using intermittent hypoxia models will be needed to better reflect clinical scenarios. Despite the limitations of our model, our findings provide a foundation for future studies aimed at dissecting the upstream mechanisms of mitochondrial dysfunction and evaluating cardioprotective strategies. Further investigation using intermittent hypoxia models, metabolic modulation, and mitochondrial-targeted therapies would be essential to bridge the gap between experimental findings and clinical application.

## 5. Conclusions

This study provides experimental data that subacute hypoxia induced by a tracheal stenosis model leads to significant cardiac remodeling and mitochondrial dysfunction via apoptotic pathways. The findings emphasize the need for early identification and targeted interventions during the subacute phase of hypoxia to prevent progression to chronic heart damage, such as heart failure. The rabbit tracheal stenosis model could be a reliable and physiologically relevant tool for studying hypoxia-related cardiac pathophysiology and warrants an evaluation for potential therapeutic strategies.

## Figures and Tables

**Table 1:** Comparison of experimental and sham groups.

	Experiment Group		Sham Group
	Before	After	
Number	6		3
Sex	all males		all males
Age	10 weeks		10 weeks
Weight	3.21 ± 15	2.15 ± 12 *	3.25 ± 25
Grade of Stenosis (Range)	2–3	66 (53–83)	1

* *p* = 0.04 in the Wilcoxon Singed-Rank test.

## Data Availability

The datasets used and analyzed in the current study are available from the corresponding author upon reasonable request.

## Supplementary Materials

The following supporting information can be downloaded at https://www.mdpi.com/article/10.3390/jcdd12100377/s1, Supplement Table S1. Physiological and clinical parameters baseline and before euthanasia in experimental and sham rabbits; Supplementary Figure S1. Representative echocardiographic parasternal long-axis view at baseline and 2 weeks after tube removal in a tracheal stenosis rabbit; Supplementary Figure S2. Representative echocardiographic estimates from parasternal long-axis view at baseline and 2 weeks after tube removal in a tracheal stenosis rabbit.
