# Supplementary material for: Subacute Hypoxia Induces Cardiac Remodeling and Mitochondrial Dysfunction via Apoptotic Pathways in a Rabbit Model of Tracheal Stenosis
Source: J Cardiovasc Dev Dis. 2025 Sep 24;12(10):377. doi: 10.3390/jcdd12100377 (PMC12564887; doi:10.3390/jcdd12100377)
Supplement: Supplementary file 1 [file jcdd-12-00377-s001.zip › jcdd-3834638-supplementary.pdf]

Supplement Table S1. Physiological and clinical parameters baseline and before euthanasia in experimental and sham rabbits.

	No.	Grade	Weight (Kg)		Stridor (Yes)		SpO_2 (%)		Respiratory rate (/min)	
			Baseline	euthanasia	Baseline	euthanasia	Baseline	euthanasia	Baseline	euthanasia
**Experiment**	1	2	3.3	2.2	0	2	97	90	30	45
	2	3	3.2	2.1	0	3	96	88	28	50
	3	2	3.2	2.2	0	2	95	89	30	44
	4	3	3.1	2.0	0	2	96	87	29	48
	5	3	3.3	2.2	0	2	97	88	35	55
	6	3	3.2	2.2	0	3	96	88	32	52
	**mean**	2.67	3.21	2.15	0	2.33	96.17	88.33	30.67	49
**Sham**	1	1	3.4	3.3	0	0	97	96	28	30
	2	1	3.2	3.1	0	0	95	94	35	36
	3	1	3.4	3.2	0	0	96	95	32	33
	**mean**	1	3.33	3.2	0	0	96	95	31.67	33

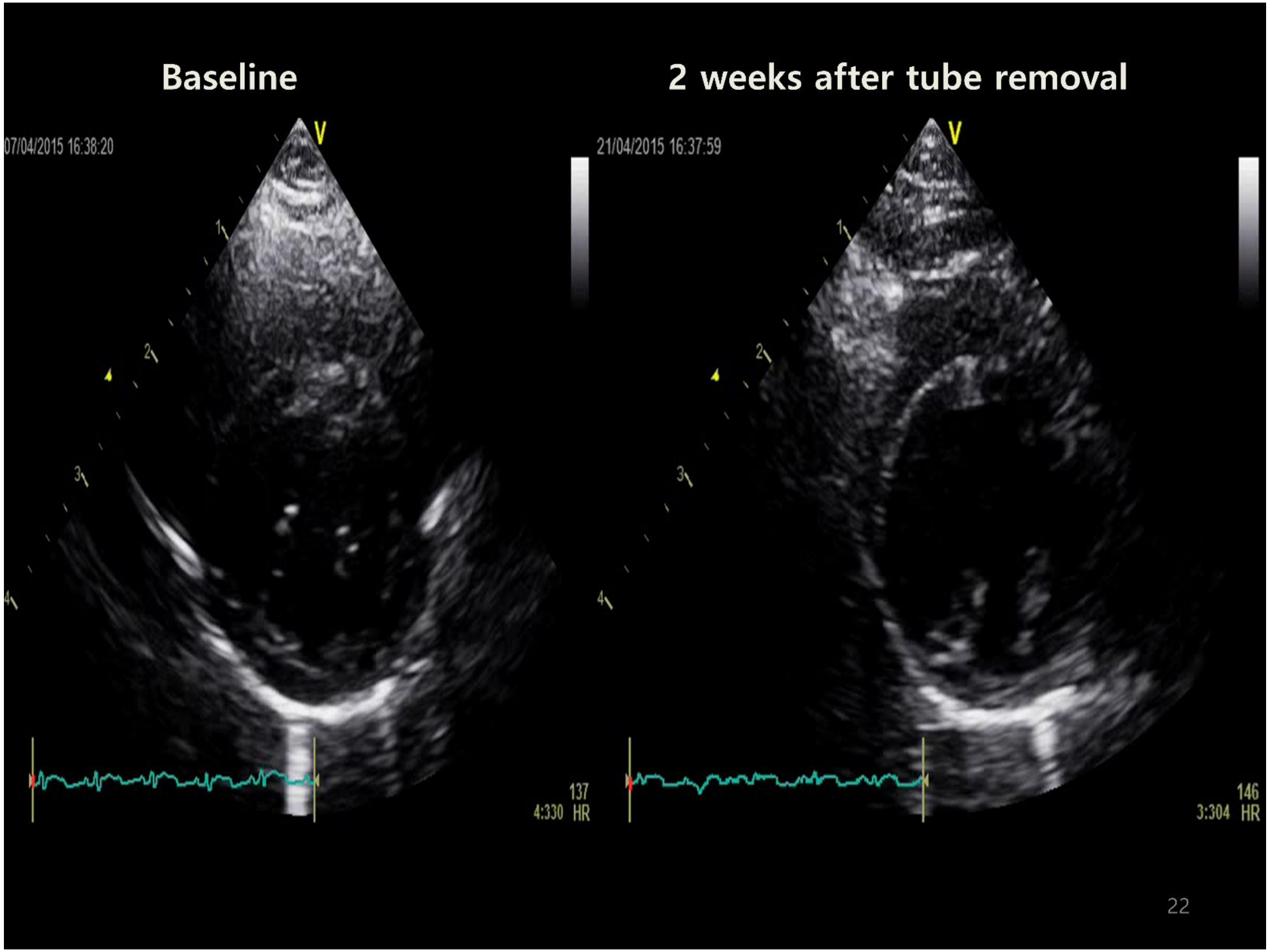

Supplementary Figure 1. Representative echocardiographic parasternal long-axis view at baseline and 2 weeks after tube removal in a tracheal stenosis rabbit.

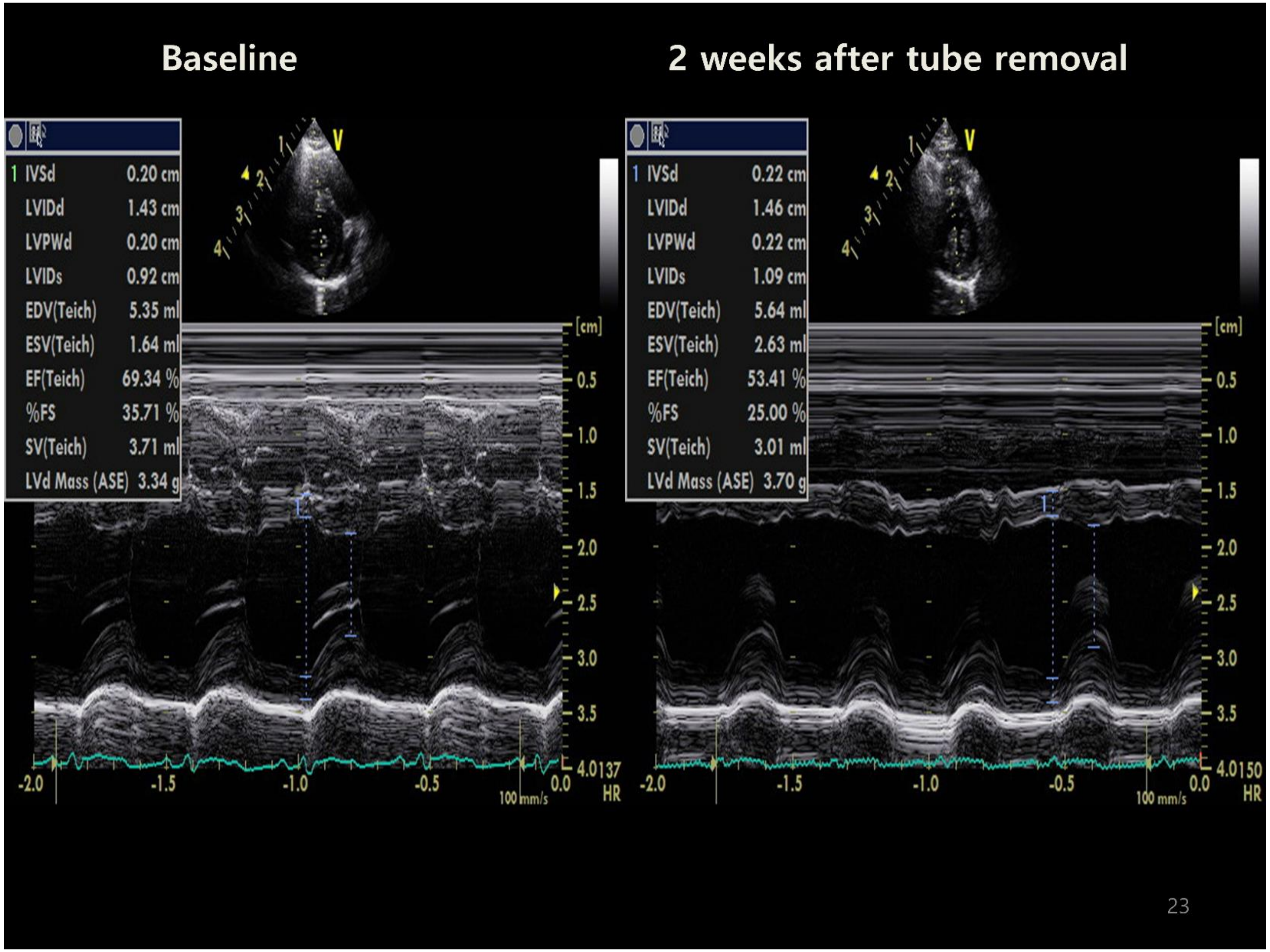

Supplementary Figure 2. Representative echocardiographic estimates from parasternal long-axis view at baseline and 2 weeks after tube removal in a tracheal stenosis rabbit.
